# Immune Thrombocytopenic Purpura in an Adult Male: A Case Report

**DOI:** 10.7759/cureus.46664

**Published:** 2023-10-08

**Authors:** Suchit Thapa Chhetri, Bishal Kunwor, Bishal Sharma, Prerana Joshi, Sunil Timilsina

**Affiliations:** 1 College of Medicine, Nepalese Army Institute of Health Sciences, Kathmandu, NPL; 2 College of Medicine, Gandaki Medical College, Pokhara, NPL; 3 General Practice and Emergency Medicine, Shree Birendra Hospital, Kathmandu, NPL

**Keywords:** ecchymosis, blood platelet, immune, thrombocytopenic, purpura

## Abstract

Immune thrombocytopenic purpura (ITP) is an autoimmune disease characterized by immune-mediated destruction of platelets, resulting in a decreased blood platelet count (less than 100 x 10^9^/L) in the absence of other known etiology of thrombocytopenia. ITP is uncommon in adult males. The signs and symptoms of ITP vary widely and are quite diverse. The degree of thrombocytopenia and bleeding are not always correlated. Timely diagnosis, intervention, and regular monitoring can easily prevent complications. We report a case of a 22-year-old male presented with gum bleeding along with purpura and ecchymosis over the upper limb, lower limb, trunk, and face.

## Introduction

Immune thrombocytopenic purpura (ITP) is an autoimmune disorder characterized by immune-mediated destruction of platelets, resulting in a decreased platelet count (less than 100 x 10^9^/L) in the absence of other known etiology of thrombocytopenia [1]. Epidemiological studies on ITP have documented incidence rates ranging from 1.1 to 5.8 per 100,000 people among children and from 1.6 to 3.9 per 100,000 among adults [2]. ITP can be categorized into primary (idiopathic) and secondary types, with the latter being associated with other conditions such as infections, autoimmune diseases, or drug exposure [1].

ITP presents with clinical features such as purpura, petechiae, and mucosal bleeding [3]. The pathogenesis of ITP involves autoantibodies (primarily Immunoglobulin G) targeting platelet surface antigens, leading to their destruction by phagocytic cells, particularly in the spleen, with immune dysregulation [4]. Clinical observation and experience indicate a spectrum of manifestations ranging from trivial bruising to catastrophic hemorrhage [5].

This case report presents a unique case of ITP, focusing on the clinical features observed, the diagnostic evaluation conducted, and the treatment strategies employed. By documenting this case, we contribute to the existing knowledge of ITP and provide insights into its management.

## Case presentation

A 22-year-old male without known prior co-morbidities presented to the Emergency Department with complaints of gum bleeding for one day and maculopapular skin rashes over the upper limb, lower limb, trunk, and face for four days. The patient denied the use of any medication and history of recent trauma and allergy. He was a chronic smoker (pack year: 2) and chronic alcohol consumer (12 units/day). There was no history of familial bleeding disorders. There was no history of hematuria, melena, hemoptysis, epistaxis, joint swelling, pain in the abdomen, headache, abnormal body movement, and chest pain. There was no history of blood transfusion in the past.

On examination, the vitals were normal and the patient was hemodynamically stable. The findings of the systemic examination were unremarkable. Upon admission to the Emergency Department, the patient's vital signs were recorded as follows: blood pressure, 118/76 mm Hg; heart rate, 78 beats per minute; respiratory rate, 15 breaths per minute; and temperature, 37.6°C. On head-to-toe examination, multiple purpura and ecchymosis were seen over the chest, abdomen, face, and extremities indicating a systemic cause. Laboratory tests were ordered on the spot (Table 1). 

**Table 1:** Important Laboratory Results of the Patient

Investigation	Result	Reference Range
Platelets	7000 cells/mm^3^	150,000-400,000 cells/mm^3^
Red blood cells (RBC)	4.10 million/mL	1.5-4.5 million/mL
Total lymphocyte count (TLC)	9410 cells/mm	4000-11,000 cells/mm
Hemoglobin	14.9 g/dL	11-16 g/dL
Prothrombin time	14 s	12-16 s
International normalized ratio	1	1

**Figure 1:**
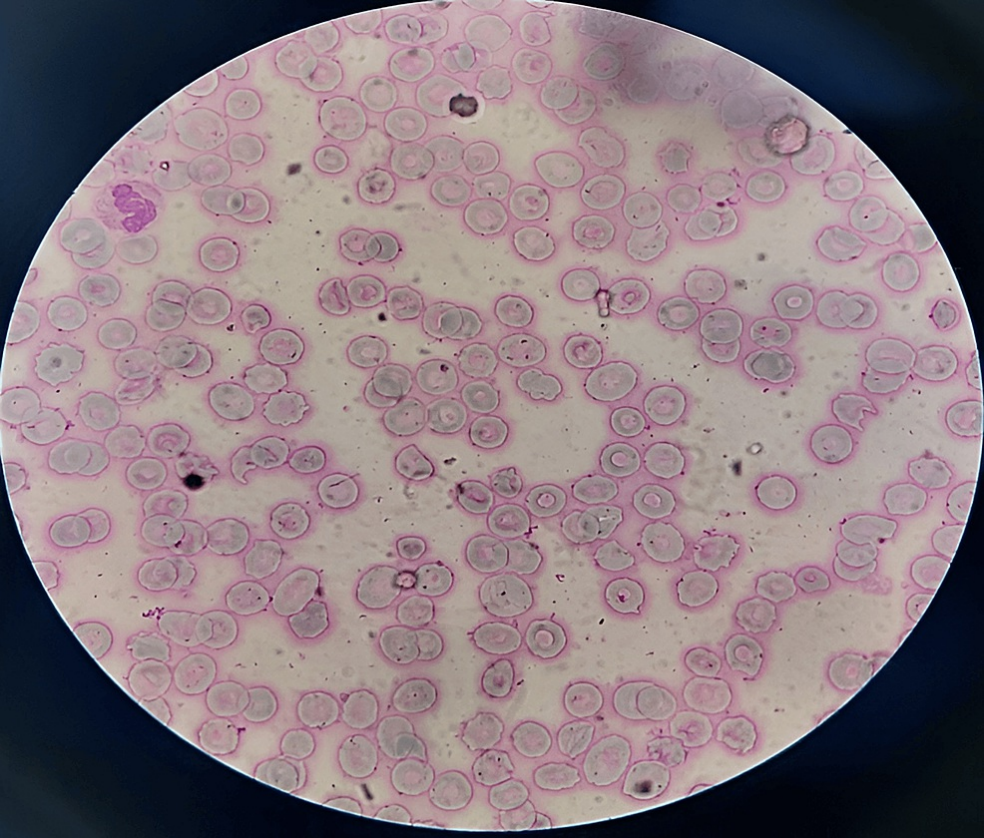
Smear showing no platelets in this field with normocytic normochromic RBC and one neutrophil in the upper left quadrant.

Blood tests revealed a normal liver function test (LFT), renal function test (RFT), and a normal coagulation screen. The platelet count was 7000 cells/mm^3^ of blood, which was severely low with normal bleeding time, prothrombin time, and partial thromboplastin time. The D-dimer, fibrinogen, and fibrin degradation products were all negative with a normal range of sedimentation rate and C-reactive protein. Peripheral blood smear (PBS) showed a decreased platelet count (thrombocytopenia) with normal RBC and WBC morphology and absence of atypical cells and hemoparasites (Figure 1). 

Immunochromatography of blood for HIV I and II, HBsAg, and HCV antibody tests were non-reactive. On stool examination for *Helicobacter pylori*, the finding was negative. Ophthalmological examination showed no retinal hemorrhages. Ultrasonography (USG) of the abdomen and pelvis did not reveal any significant findings. No findings suggestive of disseminated intravascular coagulation (DIC) were detected.

**Figure 2:**
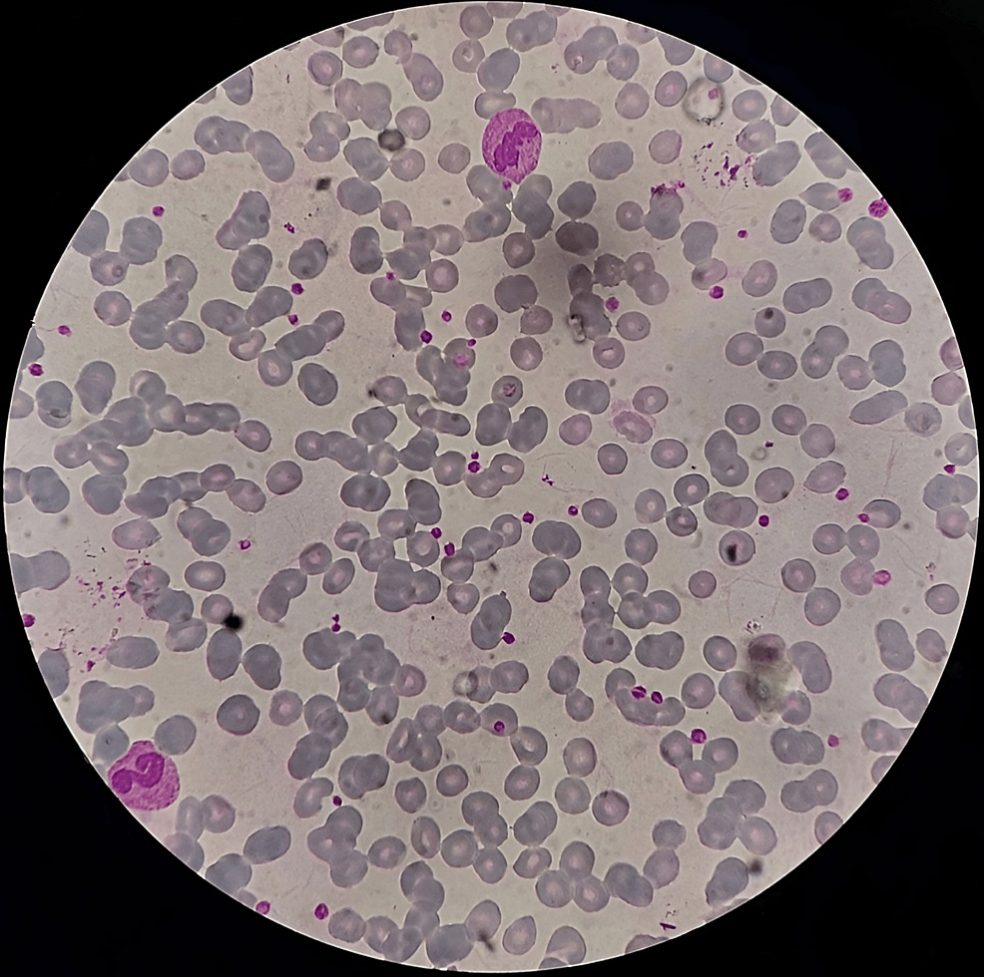
Smear showing an adequate number of platelets at the time of discharge.

A diagnosis of primary ITP was made in the absence of secondary causes. The patient was transfused with 8 pints of platelet-rich plasma (PRP) and managed with IV methylprednisolone during the entire duration of stay in hospital. The platelet count in the subsequent complete blood count (CBC) improved from 7000 cells/mm^3^ to 9000 cells/mm^3^ to 13,000 cells/mm^3^ to 70,000 cells/mm^3^ to 200,000 cells/mm^3^ (Figure 2). The patient was then discharged and instructed to take Tab. Methylprednisolone PO for seven weeks in doses of 60 mg, 50 mg, 40 mg, 30 mg, 20 mg, 10 mg, and 5 mg each week, along with Tab. Esomeprazole 40 mg PO for two weeks. The patient was suggested to avoid strenuous exercise and advised to regular follow-up with CBC report. Bleeding complications were not observed after discharge and platelet levels were seen normal during follow-up.

## Discussion

ITP is caused due to immune-mediated platelet destruction. A study done by Schoonen et al. found that the incidence of ITP is 3.9 per 100,000 patient-years, with a higher rate in women (4.4 per 100,000 person-years) compared to men (3.4 per 100,000 person-years) [6]. In males, there is a bimodal distribution, with the highest rates in boys under 18 and men aged 75-85 years while for females, incidence rates remain relatively constant from childhood to age 60, after which they increase [6]. Similarly, Moulis et al.'s study, found an overall incidence rate of 2.9 per 100,000 patient-years, with peak incidences occurring in childhood (1-5 years) and among the elderly (>60 years) [7]. While overall rates were higher in women, there were notable incidence peaks among young boys (0-5 years) and older men (>75 years) [7]. In general, ITP tends to be more prevalent in females overall, except for instances of higher incidence among boys in early childhood and older men (>75 years).

Diagnosis of ITP requires the exclusion of other etiologies for isolated thrombocytopenia [8]. The American Society of Hematology states that the history, physical examination, complete blood count, and peripheral smear examination are the main factors in making the diagnosis of ITP [9]. ITP is classified into three categories based on duration: newly diagnosed, persistent (3-12 months), and chronic (12 months) [1]. In our case, the phase of the patient was compatible with newly diagnosed ITP.

The signs and symptoms of ITP vary widely and are quite diverse. ITP can present as asymptomatic cases with slight bruising and mucosal bleeding, such as oral or gastrointestinal hemorrhage, or as frank bleeding from any site [10]. A study showed that the adult population with ITP presented with the following bleeding symptoms: purpura (62.8%), gingival bleeding (19.9%), epistaxis (10%), hematuria (6.6%), melena (3.8%), menorrhagia (3.9%), cerebral bleeding (0.7%), and other bleeding symptoms (3.1%) [11]. Our case had symptoms of purpura and gingival bleeding.

In the absence of extenuating factors that increase the risk of major bleeding, immediate therapy is not required in patients with platelet counts of more than 20,000 cells/mm^3^ [12]. However, patients with platelet counts less than 20,000 cells/mm^3^ to 30,000 cells/mm^3^ and also those with counts less than 50,000 cells/mm^3^ with substantial mucous membrane bleeding (or bleeding risk factors including hypertension, peptic ulcer disease, or an active lifestyle) should receive treatment. Treatment regimens include high-dose parenteral glucocorticoid therapy, intravenous immunoglobulin G, and platelet transfusion [12]. Splenectomy is indicated in patients with bleeding symptoms if platelet counts remain below 30,000 cells/mm^3^ after four to six weeks of medical treatment.

## Conclusions

This case described an adult male patient presenting with symptoms of gum bleeding with purpura and ecchymosis over the body. Diagnosis of ITP was made through clinical presentation, a low platelet count on CBC, and exclusion of other potential causes of thrombocytopenia. This case underscores the need for thorough diagnostic evaluation when facing isolated thrombocytopenia and highlights the varied clinical manifestations of ITP. This report also highlights a unique aspect of ITP as it pertains to the male patient demographic. It underscores the importance of recognizing the varying age and gender-related patterns in ITP presentations. It also emphasizes the importance of individualized treatment based on platelet counts and bleeding symptoms. ITP is a rare condition that can have serious complications if not treated promptly. Timely diagnosis, intervention, regular monitoring, and follow-up are essential to ensure the patient's recovery and prevent complications. This case highlights the need for increased awareness and tailored management strategies for ITP, particularly among the adult male population, and underscores the importance of individualized care.
